# Are Changes in Corticomotor Excitability Associated with Improved Arm Functional Performance Following a Tailored Strength Training Intervention in Chronic Stroke Survivors?

**DOI:** 10.3390/brainsci15070700

**Published:** 2025-06-29

**Authors:** Stephania Palimeris, Yekta Ansari, Anthony Remaud, François Tremblay, Hélène Corriveau, Marie-Hélène Boudrias, Marie-Hélène Milot

**Affiliations:** 1 Faculty of Medicine and Health Sciences, School of Physical and Occupational Therapy, McGill University, Montréal, QC H3G 1Y5, Canada; stephania.palimeris@mail.mcgill.ca (S.P.); mh.boudrias@mcgill.ca (M.-H.B.); 2 BRAIN Lab, Jewish Rehabilitation Hospital, CISSS-Laval, Laval, QC H7V 1R2, Canada; 3 Montreal Center for Interdisciplinary Research in Rehabilitation (CRIR), Montréal, QC H3S 1M9, Canada; 4 Bruyère Research Institute, Ottawa, ON K1R 6M1, Canada; yansa084@uottawa.ca (Y.A.); aremaud@bruyere.org (A.R.); ftrembla@uottawa.ca (F.T.); 5 Faculty of Health Sciences, School of Rehabilitation Sciences, University of Ottawa, Ottawa, ON K1S 5S9, Canada; 6 Faculté de Médecine et des Sciences de la Santé, École de Réadaptation, Université de Sherbrooke, Sherbrooke, QC J1H 5N4, Canada; helene.corriveau@usherbrooke.ca; 7 Centre de Recherche sur le Vieillissement, CIUSSS de l’Estrie-CHUS, Sherbrooke, QC J1H 4C4, Canada

**Keywords:** stroke, arm strengthening exercises, tDCS, motor threshold, MEP, silent period

## Abstract

Background/Objectives: We showed that a tailored strengthening intervention based on the size of motor evoked potentials (MEPs) elicited by transcranial magnetic stimulation (TMS) in the affected hemisphere resulted in an improved affected arm function, regardless of stroke severity. Also, adding anodal transcranial direct stimulation (atDCS) during training did not alter the results as participants receiving real or sham stimulation showed similar gains. The goal of this study was to report on the changes in basic measures of corticomotor excitability in response to the intervention and to determine whether these changes were influenced by tDCS and correlated with those measured in arm function. Methods: The TMS measures consisted of the resting motor threshold (rMT), MEP amplitude at rest, and the silent period (SP) duration. Clinical outcomes included the Box and Block test (BBT) and grip strength (GS). Results: Post-intervention, regardless of atDCS (*p* > 0.62), no significant change in corticomotor excitability was noted (*p* > 0.15), as well as no association between the changes in TMS measures and arm function gains (*p* > 0.06). Conclusions: As observed for clinical measures, atDCS did not influence corticomotor excitability. The absence of an increase in the excitability of the affected hemisphere and important associations between changes in corticomotor excitability and clinical gains suggest that factors other than brain plasticity could mediate gains in arm function. Further investigations are required regarding the role of tDCS in stroke rehabilitation.

## 1. Introduction

It is estimated that about 101 million people worldwide are living with the consequences of a stroke [1]. Following initial damage, the neuroplasticity process is initiated in brain circuits to compensate for both the lesion itself and its remote effects [2]. Neuroplasticity is defined as the brain’s capacity to undergo functional and structural changes through growth and reorganization in the residual surviving brain tissue [3,4]. Although most stroke survivors experience some degree of recovery, many are left with impairments in the long term that lead to activity limitations and restrictions in participation [5]. For instance, more than three-quarters of stroke survivors present with lingering muscle weakness in the affected upper limb (UL), leaving them with poor arm function and a huge impact on their quality of life [6]. Thus, improving the arm function is of critical importance for chronic survivors. One intervention to counter residual weakness and improve arm function after a stroke is through strengthening exercises [7,8]. Yet, patients often show variable responses to training; some individuals show significant gains while others show either minimal or no benefits [9,10]. As we have argued before [11,12], this variable response to strength training likely reflects the fact that exercise interventions are often provided in bulk and not properly tailored for an individual’s capacity in terms of frequency and intensity [13,14,15].

Motor evoked potentials (MEPs) elicited by transcranial magnetic stimulation (TMS) of the affected hemisphere not only provide a valid marker of corticospinal tract integrity following a stroke, but also provide insights into the potential for recovery [5,16,17,18]. Several measures of corticomotor excitability can be derived from MEPs recordings in the extremities. First, the size of MEPs can provide an assessment of the excitability at both the cortical and spinal level, the amplitude being a reflection of the indirect activation of corticospinal neurons [19]. Second, the resting motor threshold (rMT), which is determined as the minimal intensity to elicit detectable MEPs at rest, provides insights into the excitability of neuronal elements at the core of the motor representation in the motor cortex. The silent period (SP) is another basic measure of corticomotor excitability that is measured when TMS pulses are delivered during active contraction of the target muscle. The SP is detected as an interruption in the ongoing muscle activity after the TMS pulse, and its duration (especially the later portion) provides insights into the excitability of cortical interneurons mediating inhibition via GABAb receptors activation [5,19,20]. TMS investigations have revealed several abnormalities reflecting reduced corticomotor excitability in the affected hemisphere of stroke survivors, such as small amplitude or absent MEP responses, increased rMTs, and prolonged SP durations [21,22,23,24]. Although changes in TMS outcome measures often parallel gains in clinical outcome measures following training, a paucity of studies has directly assessed the association between training-induced changes in neurophysiological and behavioral outcomes in stroke, with mitigated results [5,25,26]. For example, following robotic training of the UL in subacute stroke survivors, Sehle et al. found a positive association between gains in upper extremity Fugl-Meyer Assessment (UE-FMA) and changes in MEP amplitude (r = 0.43; *p* < 0.04), but not with SP [26]. In chronic stroke survivors, Koski et al. reported a significant positive association between changes in rMT and gains in motor function (UE-FMA: r = 0.71; *p* = 0.03) and functional ability (Wolf motor function test: r = 0.69; *p* = 0.04) following UL training tailored to each participant’s goals [25].

Previously, we sought to determine whether tailoring strengthening exercises targeting the UL using MEPs as a marker of corticospinal integrity in the affected hemisphere could lead to more optimal rehabilitation outcomes. Our results showed that this approach was largely effective in improving the function and performance of the affected UL, regardless of the stroke severity in a large cohort of participants [12]. These results provided further evidence of the clinical relevance of exercise intervention in leading to improved arm function in stroke survivors even at the chronic stage [12,27]. In recent years, noninvasive brain stimulation (NIBS) techniques have been highlighted as a promising tool to promote adaptive plasticity mechanisms [28,29]. NIBS techniques, such as transcranial direct current stimulation (tDCS), aim to modulate cortical excitability, which can putatively help to enhance recovery [30]. However, studies that have evaluated the potential impact of tDCS combined with rehabilitation interventions have produced mixed results so far [12,31,32,33,34,35]. At the functional level, some studies have highlighted the add-on effect of tDCS on post-stroke arm function gains following various rehabilitation interventions [36], while other studies, like our MEP-based tailored UL training study [12], showed no impact of tDCS on post-training arm function [33,37]. At the neurophysiological level, some studies have found an increase in corticomotor excitability, such as an increase in MEP amplitude or a decrease in rMT, following rehabilitation interventions with tDCS [32,34,35], while others showed no benefit of tDCS [32,33,35].

In the present report, we describe our observations regarding the changes in basic measures of corticomotor excitability that were recorded in response to the intervention in our large cohort of stroke survivors. In doing so, we were also interested in determining whether tDCS applications might have exerted any subtle modulatory effects that could have affected excitability measures, while being undetectable at the clinical level, as we mentioned earlier. Finally, we also wanted to determine whether changes in corticomotor excitability of the affected hemisphere could be associated with the gains in arm functional performance that were observed in our participants post-intervention.

## 2. Materials and Methods

The study was approved by the Research Ethics Committee (REC) of the CIUSSS de l’Estrie-CHUS (MP-22-2016-630) and the Bruyère Research Ethics Committee (Protocol #M16-16-028), and all participants signed a consent form prior to the study participation. The study was registered in ClinicalTrials.gov (#NCT02915185) on 22-09-2016.

The methods were described in detail in a previous paper [11]. Briefly, the participants (1st unilateral stroke at the chronic stage, >6 months) underwent clinical and neurophysiological evaluations before and in the week post-training by evaluators blinded to the participants’ tDCS allocation group.

### 2.1. Clinical Assessment

The main clinical outcome measures retained for this study were functional performance of the affected arm, evaluated with the Box and Block test (BBT: number of blocks in 60 s) [38], and grip strength (GS: average of three trials in kg). Performance in each test was determined by averaging individual trials in each tDCS group before and after the intervention.

### 2.2. TMS Assessment

The TMS assessments were performed at three different sites using a standardized protocol relying on similar equipment and materials. While participants were comfortably seated, TMS was applied using a Magstim 200^2^ stimulator (Magstim Company, Dyfed, UK) connected to a focal coil (70-mm loop). Each site used the same belly-tendon montage to record surface activity evoked in the first dorsal interosseous (FDI) on both sides and in the extensor carpi radialis (ECR) of the affected side. At the Ottawa site, electromyographic (EMG) signals were recorded and processed using a Delsys DE-2.1 system (Delsys Inc., Boston, MA, USA), whereas the Montreal site used a Neuroline 700 system (Ambu, Glen Burnie, MD, USA), and the Sherbrooke site used a PiCO EMG Cometa system (Bareggio, Italy). At each site, the EMG signals were sampled at 2000 Hz, band-pass filtered (6–450 Hz), amplified (×1000), and saved on a PC for offline analysis.

To determine the rMT, TMS was applied first to the unaffected hemisphere to estimate the location of the hot spot for the FDI. From this estimated position, the coil was moved over the same spot on the affected hemisphere, and stimulator output was increased gradually in an attempt to evoke MEPs in the FDI. In the participants in whom MEPs could be evoked in the FDI, the rMT was then determined using the Motor Threshold Assessment Tool software (MTAT 2.0; Clinical Researcher, Knoxville, TN, USA), which allows fast estimation of rMT through the maximum-likelihood strategy based on the Parameter Estimation by Sequential Testing (PEST) algorithm [39]. The criterion to determine the presence or absence of evoked response in the affected hand was set at MEPs > 50 μV. In participants with no detectable MEPs in the FDI, the coil was slightly moved medially to target the representation of the affected ECR. If no detectable MEPs could be recorded (<50 μV), the participant’s response was deemed to have no response, i.e., “MEP absent”.

In participants with detectable MEPs and in whom the rMT could be determined, MEPs at rest were measured. For this test, the stimulator was set at 130% of the rMT or at 100% output in cases where the 130% rMT exceeded the maximal stimulator output. Then, 10 MEPs were recorded at rest.

Following testing at rest, corticomotor excitability of the lesioned hemisphere was tested in the active state to assess the SP duration. The participants were asked to exert a light pinching force on a dynamometer using a lateral key pinch (about 20% of their maximal force) with the affected thumb and index finger for 5 s. During the contraction, a suprathreshold TMS pulse (130% of rMT) was delivered at 3 s to elicit an SP. This procedure was repeated 5 times with a 30 s rest between each trial. Note that 21 participants were not able to provide a light pinching force during this evaluation; so their SP duration could not be determined.

### 2.3. Strength Training and tDCS Intervention

The strength training program was described in detail in our previous paper [11]. Briefly, after stratification based on MEP amplitude, the participants were assigned to three intensity groups [11,40]: (1) low-intensity (LI: MEPs < 50 μV); (2) moderate-intensity (MI: MEPs 50–120 uV) and (3) high-intensity (HI: MEPs > 120 uV). Within each group, the participants were then randomized to receive either real or sham tDCS. For strength training, the affected shoulder and elbow flexors, wrist extensors, and handgrip muscles were trained using free weights, starting at an intensity of 35%, 50%, and 70% of 1 RM (maximal load that can be lifted once), respectively, for the LI, MI, and HI groups. The intensity was increased each week so that participants trained at 50%, 65%, and 85% in the LI, MI, and HI groups, respectively, at week 4. Each group trained 3 times a week for a duration of 4 weeks. The handgrip muscles were trained using a Jamar^®^ hydraulic hand dynamometer. For the first 20 min of each training session, tDCS (Ottawa: HDCStim, Newronika, Milano, Italy; Montreal: NeuroConn, Llmenau, Germany; Sherbrooke: Soterix Medical, New York, NY, USA) was applied using an anodal montage targeting the hand area of M1 at an intensity of 2 mA with saline-soaked 5 × 7 cm electrodes. For the sham group, the current was ramped up for 30 s and then ramped down for another 30 s [41] to simulate the real stimulation. All participants were naïve to tDCS.

### 2.4. TMS Data Analysis

Data extracted from the TMS assessment of the affected hemisphere were analyzed offline to derive individual values. The rMT was expressed in terms of percent maximum stimulator output (% MSO). MEP amplitude was determined by overlaying successive trials (n = 10) to obtain a mean peak-to-peak amplitude in µV for each participant. The SP duration was determined on a trial-by-trial basis following the procedure of Davidson et al. [42]. In each trial, the SP was defined as the time interval between the onset of the MEP and the sustained return (>50 ms) of EMG activity. Trials were averaged to obtain a mean SP duration.

### 2.5. Statistical Analysis

Participants showing no MEP (n = 10), having an rMT of 100% (n = 8), and an SP < 50 ms (n = 2) were discarded from the analysis. Sociodemographic characteristics between the real and sham tDCS groups were compared using independent t-tests and chi-squared tests. The normality of the TMS data was tested using the Shapiro–Wilk test, whereas the presence of outliers was tested using the Grubbs test. Since the data were not normally distributed and presented outliers, a log transformation was applied. To determine the impact of the intervention and tDCS on measures of corticomotor excitability, each dependent variable (i.e., rMT, MEP amplitude, SP duration) was entered into a repeated measures analysis of variance with ‘Time’ as the repeated factor (pre, post) and tDCS as the between-subjects factor (real, sham). Upon detection of main effects or interactions, post-tests were performed using the Bonferroni test. To examine possible associations between variables, each variable was first transformed into differences (post-pre). For rMT and SP, the post-pre differences were then converted into positive values, since a decrease in these values is considered an improvement, as opposed to clinical gains. Then, Pearson’s moment correlation coefficients were computed. The significance level was set at 0.05 for all tests.

## 3. Results

### 3.1. Participants Characteristics

Table 1 presents the sociodemographic characteristics of the participants. No baseline difference was noted between the two tDCS groups, except for handedness.

### 3.2. Variations in TMS Measures with the Intervention

For rMT, no significant effect of time (*p* = 0.15) was noted with no “tDCS group X Time” interaction (*p* = 0.69). For the mean MEP amplitude, no effect of time was noted (*p* = 0.63) with no “tDCS group X Time” interaction (*p* = 0.87). For the SP, no effect of time (*p* = 0.15) as well as no “tDCS group X Time” interaction (*p* = 0.62) was observed (see Table 2).

### 3.3. Association Between Changes in TMS Outcome Measures and Changes in Arm Function

**Figure 1:**
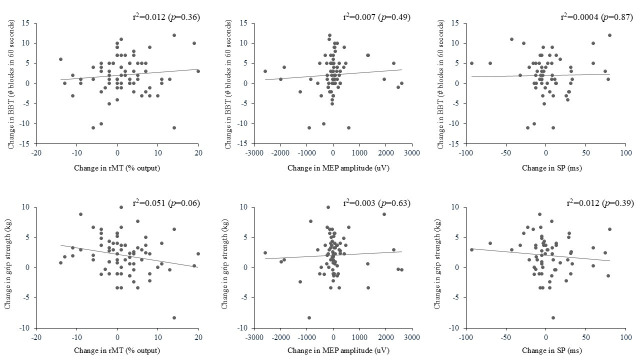
Association between changes (post-pre) for TMS and arm clinical outcome measures. Positives values on the X and Y axis are towards an improvement.

Because adding tDCS to our tailored strengthening intervention did not impact the changes in motor cortex excitability, the association between changes in TMS outcome measures and arm functional performance were analyzed without considering tDCS allocation of participants. As seen in Figure 1, no significant association between the changes in all TMS outcomes and arm clinical outcomes was found.

## 4. Discussion

This study demonstrates that adding anodal tDCS to a tailored MEP-based UL strength training program had no add-on value to enhance brain excitability. Also, gains in arm function were not associated with changes in basic TMS measures.

### 4.1. Effects of the Intervention on TMS Outcome Measures

Adding anodal tDCS to the MEP-based tailored UL strength training program did not increase participants’ brain excitability for our stroke participants. These results align with our previous observation that no benefit of tDCS was noted in gains in UL function post-training for the same cohort [12]. It was speculated that the tailored training program successfully improved UL function, regardless of baseline stroke severity, leaving no room for tDCS to have any add-on value in this improvement. This could also be the case for brain excitability measures.

This study is not the first to face a lack of tDCS effects on TMS measures. In both healthy and stroke populations, numerous studies have reported no add-on effects of anodal tDCS on various TMS measures [33,35,43,44] or a moderate one, mostly for MEP amplitude or rMT [32,34,45]. Although our tDCS protocol followed standard guidelines for stroke, its application remained generic. A more tailored application is now preconized to optimize tDCS response in stroke, which considers each neuroanatomical characteristic’s influence [46]. Also, the individual differences in brain sensitivity to TMS have been shown to influence the efficacy of tDCS in healthy participants: those with lower rMT (higher brain sensitivity to TMS) tend to respond better to tDCS [47]. However, we did not observe this phenomenon in our cohort of stroke participants. Finally, the effects of tDCS are thought to be confined to intracortical neurons [48,49], while TMS measures, such as rMT and SP, seem to depend on cortical and corticospinal neuron polarization [8,50]. This suggests that the effects of tDCS may not have been sufficient to induce a relevant shift in TMS measures, which could explain its lack of effects on brain excitability. Overall, in the chronic stage of a stroke, the impact of this modality on brain excitability is still not fully understood and requires further investigation to support its use in stroke rehabilitation.

Our MEP-based tailored strength training program did not translate into a significant increase in brain excitability, as opposed to significant changes in arm functional performance [12]. It is surprising, knowing that high-intensity training, such as in our intervention, mostly drives neurological rather than muscular adaptations [51]. It could be thought that strength training may have led to adaptations at lower levels of the motor system, such as the spinal circuits, rather than the motor cortex, that could not be detected by single-pulse TMS. Thus, even if arm functional performance increased with training, corticomotor excitability might not show dramatic changes [52,53]. Also, a high intersubject variability in TMS responses is noted in the literature [54], especially for a population with a neurologic condition [5]. As such, all our TMS outcomes did improve with training, but the high intersubject variability we observed could have prevented the detection of significant changes in our TMS measures with training. The fact that training did not impact measures of corticomotor excitability also corroborates other studies, where inconsistent changes in TMS results are reported [5,7,24,53,55]. Consequently, in chronic stroke, TMS might not be the best tool to assess neuroplasticity associated with strength training [52], as it might be able to capture only important, not subtle, neural changes. Other imaging techniques (e.g., magnetic resonance imaging [MRI] or diffusion tensor imaging [DTI]) might prove more useful in detecting neuroplastic changes with exercises in stroke [53].

### 4.2. Association Between Changes in TMS Outcome Measures and Arm Functional Performance

The arm functional gains noted post-intervention were not associated with any changes in TMS outcome measures, even though BBT and GS force production are directly linked to corticospinal integrity in stroke [56,57,58]. This is not in line with studies reporting positive associations between TMS measures [5,25,26] and clinical gains following various rehabilitation interventions in stroke. For example, in the study by Koski et al. [25], a positive correlation between changes in rMT and arm motor ability performance was noted (*R*^2^ = 0.47; *p* = 0.04). Since our training program targeted the entire UL and not solely the hand, as in Koski et al.’s study, this could have limited our impact on the change in basic TMS measures of the affected FDI. On the other hand, several studies have found no association between gains in arm function and changes in corticomotor excitability [5,7,26]. The reasons could be that stroke individuals can use various compensatory mechanisms to achieve functional gains with training, not all of which involve increased corticomotor excitability [58,59]. Also, activation or reorganization of other motor pathways, not targeted by our TMS evaluation, might have occurred in parallel with gains in arm function [60]. In addition, these results suggest that promotion of brain repair and recovery at the spinal level, using various techniques such as cell therapy, could be a promising avenue for addressing long-term disability after a stroke [61]. Overall, TMS measures can serve as an index for predicting better responders to training [40], but, as observed here, changes in TMS measures cannot provide an index of the amount of gains in arm functional performance derived from training in chronic stroke survivors.

### 4.3. Limitations

One limitation of the study can be attributed to a possible lack of consistency in the data collection, as it took place in three different sites in Canada, although the research personnel were all trained beforehand to decrease inconsistencies. In addition, not all sites had the same material (e.g., tDCS machine). There is also a limitation in the selection criteria, as individuals with major impairments post-stroke who could not perform the training program were excluded from the study. This, in addition to the multiple exclusion criteria, limits the generalizability of the results to the overall chronic stroke population. Also, stroke size and location were not collected in this study. Knowing their influence on recovery, analyzing our data based on these variables could have provided relevant information on the participants’ responses to the protocol. Considering TMS limitations, the extent or spread of the induced current in the brain may be variable. In essence, it is impossible to precisely determine the surface of cortical tissue, or the number of cortical areas stimulated with each TMS pulse. Also, the effects of the TMS on these neurons could be excitatory, inhibitory, or state-dependent [62]. In addition, we did not evaluate the map area size during the TMS evaluation because of time constraints. However, map area size might be a crucial variable to consider in predicting arm function gains following an MEP-based training program, since several studies have found a positive association between enlargement in map area size and clinical gains following various post-stroke rehabilitation interventions [5]. Finally, in this study, we did not include neurophysiological measures such as MRI and DTI, which could have allowed a more precise evaluation of the potential neurological adaptations following the training program combined with tDCS. It is therefore possible to assume that our protocol could have effects that are not detectable with a simple TMS measurement.

## 5. Conclusions

Following a 4-week tailored upper extremity training program based on the participants’ MEP amplitude, no enhancement in brain excitability was noted, as opposed to arm clinical gains. Also, no association between the gains in arm performance was found with changes in brain excitability. Mechanisms other than neuroplasticity could have taken place to allow gains in arm function, and this requires exhaustive investigation. Also, using tDCS combined with the exercise program did not enhance corticospinal excitability. Further research is necessary to elucidate the role of tDCS and its potential to augment the effects of strength training interventions on brain plasticity in chronic stroke survivors.

## Figures and Tables

**Table 1:** Participants’ sociodemographic characteristics.

	tDCS Real (N = 36)	tDCS Sham (N = 36)	All (N = 72)	*p* Value
Age (years, Mean ± SD)	64(12)	67(10)	65(11)	0.39
Handedness (N, right/left)	31/5	36/0	67/5	0.02
Gender (N, male/female)	24/12	23/13	47/25	0.80
Time since stroke (years)	5(3)	6(4)	6(4)	0.12
Type of stroke (N, I/H/O)	31/3/2	29/7/0	60/10/2	0.16
Side of stroke (N, right/left)	16/20	23/13	39/33	0.09

I = ischemic; H = hemorrhagic; O = other; tDCS = transcranial direct current stimulation.

**Table 2:** Pre/post changes in TMS outcome measures (mean (SD); [range]).

	Real tDCS Group		Sham tDCS Group	
	Pre	Post	Pre	Post
rMT (% stimulator output)	51 (16) [24–85]	49 (15) [28–94]	49 (14) [25–90]	47 (12) [28–71]
MEP amplitude (uV)	494 (626) [29–2979]	523 (753) [53–3486]	628 (863) [28–4372]	666 (1011) [21–4331]
SP (ms)	164 (66) [58–389]	159 (61) [64–358]	155 (72) [60–351]	154 (89) [44–436]

rMT: resting motor threshold; MEP: motor evoked potential; Sp: silent period.

## Data Availability

Dataset is available on request from the authors. The data are not publicly available due to [ethical reasons, because our ethics board was not informed that we would make the data available].
